# Chemoradiotherapy as a pivotal therapeutic modality for inoperable intrathymic thyroid carcinoma: A case report and literature review

**DOI:** 10.1097/MD.0000000000044836

**Published:** 2025-10-03

**Authors:** Xiaotong Huang, Bo Li, Mingjin Xu, Yanchen Gong, Hui Feng, Xiaojun Wang, Xintong Wang, Ye Tan

**Affiliations:** a Department of Radiation Oncology, The Affiliated Hospital of Qingdao University, Qingdao, Shandong Province, China; b Department of Medicine, Qingdao University, Qingdao, Shandong Province, China; c Department of Otolaryngology, The Affiliated Hospital of Qingdao University, Qingdao, Shandong Province, China.

**Keywords:** chamotherapy, intrathyroid thymic carcinoma, neoplasms, radiation therapy, thyroid gland

## Abstract

**Rationale:**

Intrathyroid thymic carcinoma (ITTC) is a rare group of low-grade malignant tumors, for which surgery is the treatment of choice. However, there is little evidence on the effectiveness of radiotherapy and chemotherapy in the treatment of ITTC.

**Patient concerns:**

A 50-year-old man presented with a 6-month history of a neck obstruction sensation. Clinical examination revealed a firm, fixed mass in the right thyroid lobe.

**Diagnoses:**

Imaging and biopsy confirmed locally advanced, unresectable ITTC with tracheal and esophageal invasion. Diagnosis was supported by immunohistochemistry (CD5+, CD117+).

**Interventions:**

Given the unresectable nature of the tumor, the patient was treated with definitive chemoradiotherapy. This consisted of induction chemotherapy with a docetaxel and cisplatin regimen, followed by concurrent chemoradiotherapy.

**Outcomes:**

Treatment achieved a complete response. The patient remained disease-free at 40-month follow-up.

**Lessons:**

Multimodal regimens with chemoradiotherapy backbone may achieve durable remission for unresectable ITTC, providing an alternative when surgery is contraindicated.

**Key points**

- There is currently no standardized criterion for the treatment of ITTC.
- The role of radiotherapy and chemotherapy in the treatment of inoperable ITTC remains unclear.
- The efficacy in radiotherapy and chemotherapy is unclear.
- Surgical treatment may be not the only effective way to cure ITTC.
- Combined radiotherapy and chemotherapy can effectively treat unresectable locally advanced ITTC.
- Induction platinum-based chemotherapy contributed to rapid tumor shrinkage and symptoms relief in inoperable ITTC patients.

## 1. Introduction

Intrathyroidal thymic carcinoma (ITTC) was first identified and documented by Miyauchi^[1]^ in 1985, with an incidence rate of <0.1% and >80% 10-year survival rate. It is classified as “Intrathyroidal thymic neoplasms” in the World Health Organization Classification of Tumors (5th edition, 2022; *International Classification of Diseases for Oncology* code: 8589/3, *ICD-11* code: 2C27.0*).^[2]^ ITTC is hypothesized to originate from ectopic thymic remnants or branchial pouch derivatives showing thymic differentiation,^[3]^ consisting of thyroid carcinoma with thymus-like characteristics, carcinoma showing thymus-like differentiation, and primary thyroid thymoma. ITTC is a group of low-grade malignant tumors^[4]^ with an insidious onset and potentially invasive behavior. And the invasion of peripheral organs often occurs before diagnosis.^[5]^

Currently, surgery is considered the first option for ITTC treatment, with chemotherapy and radiotherapy only in an adjunctive position.^[6]^ As a relatively high incidence of local recurrence, most experts believe radical resection is safer in contrast to radical radiotherapy. Nowadays, chemotherapy and radiotherapy seem to be effective against ITTC only in the following 3 situations: for postoperative adjuvant therapy to reduce the recurrence risk or for whom with postoperative risk factors^[7–12]^; for patients with inoperable factors or with recurrence/metastases^[13,14]^; and for palliative care to control or prevent symptoms caused by the tumor.^[15]^

The present study reported an inoperable ITTC patient who finally achieved partial remission after chemoradiotherapy. Based on which, we subsequently conducted a literature search in PubMed using the term “ITTC” and “Intrathyroid thymic carcinoma,” and discovered a controversial efficacy of chemoradiotherapy for ITTC. This study adds to the limited evidence on the efficacy of chemotherapy in the treatment of ITTC.

## 2. Case report

A 50-year-old man presented to the emergency surgery department of our hospital on November 30, 2021 with a complains: “6-month history of foreign body obstruction in the neck.” No specific past medical history occurred in this person. At the same time, he has no family hereditary disease history and no unhealthy habits such as smoking or drinking. A board-certified endocrinologist (≥10 years’ experience) revealed a firm, fixed, non-tender mass measuring approximately 4 cm in the inferior pole of the right thyroid lobe, without palpable thrill or audible vascular bruit (Eastern Cooperative Oncology Group performance status). Serology showed anti-thyroglobulin antibody at 251.00 IU/mL, antithyroid peroxidase antibody at 492.00 IU/mL, and TG at 0.77 ng/mL. Other indicators showed no signs of abnormality. Thyroid ultrasound examination: a 4.0 × 3.4 × 2.0 cm hypoechoic mass was detected in the right lobe of the thyroid gland, with a regular morphology, poorly defined borders, and poorly homogeneous internal echogenicity. It extended toward the retrosternum and was classified as Thyroid Imaging Reporting and Data System category 4a; no specific nodule was detected in the left lobe. There were no notable and substantial swollen lymph nodes observed on either side of the neck. Chest CT scan revealed a mass in the right thyroid lobe which compressed the trachea. Initially identified as thyroid neoplasms, the patient underwent excision surgery on December 6, 2021 after ruling out distant metastasis and other primary tumors. Observed during surgery, the mass was seen to be hard and fixed, and the left lobe was significantly enlarged with a diameter of about 4 cm. The thyroid tissue infiltrated into the deep cervical musculature partially extending into the retrosternum, compressing and infiltrating the trachea, making the interstitial space around the thyroid gland unclear. In the lateral posterior part of the trachea and the anterior vertebral area, there were pieces of laminated tumorous tissue that could not be resected as the laryngeal recurrent nerves were not visible. all these pieces of evidence made the radical surgery difficult to perform, so we chose local biopsy. Pathological analysis after surgery indicated the tumor located in the right lobe of thyroid gland was moderately to poorly differentiated. Coupled with the immunohistochemical findings, the results aligned with the presence of intrathyroidal thymic adenocarcinoma (spanning 4.3 × 3 cm). It is medium-low differentiated squamous cell carcinoma with thyroid gland peritubular membrane invasion and an absence of clear nerve or vascular invasion. Immunohistochemical analysis showed: CD56 (‐), Calcitonin (‐), p40 (+), Syn (partial +), CgA (scattered +), TTF-1 (‐), Pax-8 (‐), CD5 (+), CD117 (+), p63 (+), CK5/6 (+), Ki-67 (+, 8%), PD-L1 (CPS72). Genetic testing failed to pinpoint any mutation locations, lacked corresponding sensitive drugs, had a tumor mutational load of 0.0 mut/Mb, had no somatic coding mutations, and had microsatellite instability indicating microsatellite stability.

**Figure 1.**
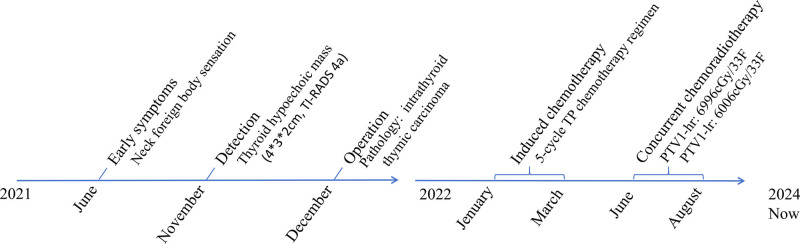
The diagnose and treatment trajectory of the ITTC patient. ITTC = intrathyroid thymic carcinoma.

**Figure 2.**
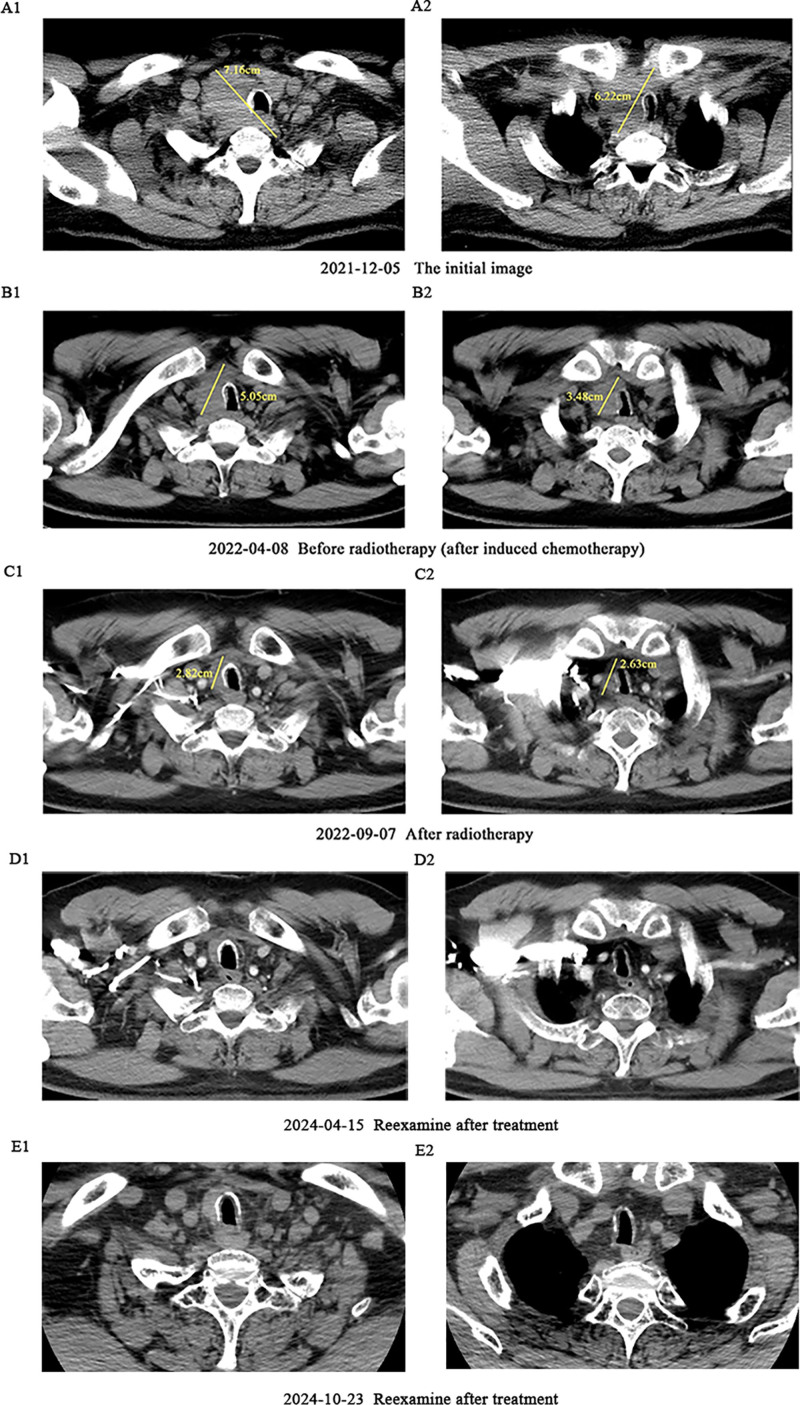
Depicts the CT image changes at the tumor level during the patient’s treatment. A1 and A2 are pre-treatment CT images; B1 and B2 are CT images after 5 cycles of chemotherapy and prior to concurrent radiotherapy; C1 and C2 are the first post-concurrent-radiotherapy review CT images; D1, D2, E1, and E2 are CT images taken at the post-treatment review. A1, B1, C1, D1, and E1 show the costal head of the first rib of the tumor, while A2, B2, C2, D2, and E2 represent the jugular notch level of the manubrium sterni of the tumor.

One month after thyroid biopsy, the patient experienced escalating chest distress and dyspnea causing unable to lie down. On January 14, 2022, a neck-enhanced CT scan revealed increased thickness in the nasopharyngeal and oropharyngeal walls, a constricted lumen, and compression of the trachea. Chemoradiotherapy was used to swiftly alleviate the patient’s symptoms of airway obstruction. Five rounds of docetaxel and cisplatin (TP) regimen induction chemotherapy (docetaxel 150 mg d1 and cisplatin 80 mg d1–2, every 3 weeks) were conducted from January 19 to May 10, 2022 (Fig. 1). The effectiveness was assessed as partial remission in the 2nd and 4th cycles, leading to notable relief in chest constriction and symptoms of airway obstruction. The patient commenced local radiotherapy on June 20, 2022, targeting the primary tumor and lymph node drainage zone, with a dosage of PTV1-hr: 6996cGy/33F, PTV1-lr: 6006cGy/33F and was treated with a single cisplatin 50 mg d1–3 chemotherapy concurrently with radiotherapy. Then, a single consolidation chemotherapy cycle using the TP regimen on September 6, 2022 was performed (Fig. 1). The tumor kept diminishing after the comprehensive treatment, and the symptoms of chest constriction and breathlessness vanished, with effectiveness assessed as complete response (CR), and no adverse and unanticipated events were found. After regular follow-up examinations, as of March 2025, the progression-free survival period is 40 months (Fig. 2).

## 3. Discussion

ITTC, a form of thyroid cancer originating from abnormal thymic tissue potentially linked to Epstein–Barr virus infection,^[16]^ is widely thought to stem from the remains of the third parotid sac during embryonic development.^[17]^ but a few studies^[18,19]^ have identified its source as the thymic duct. There are various classifications of ITTC, including ectopic malignant thymoma, ectopic cervical thymoma, thymic-like differentiated spindle epithelial tumor and thymic-like differentiated carcinoma.^[17]^ Furthermore, there are distinct tissue subtypes such as squamous, lymphoepithelioid, and neuroendocrine carcinomas. ITTC has a good prognosis, with a 5-year survival rate of 90% and a 10-year survival rate of 82% after treatment.^[7]^ Despite its low malignancy, ITTC shows significant aggressiveness, evidenced by over 50% rates of nearby tissue invasion and lymph node metastasis.^[8]^ This is similar to other poorly differentiated carcinoma of the thyroid.

ITTC has an insidious onset and a high rate of misdiagnosis, lacking clear-cut specificity in its clinical and imaging presentations.^[20,21]^ Technetium^[9]^ imaging, I131, thyroid scintigraphy, thyroid scintigraphy^[22]^ and postoperative physiology^[4,23–25]^ can support the diagnosis.

While surgery remains the primary curative modality for ITTC,^[8,26]^ its application entails significant challenges. Early-stage disease without nodal involvement may achieve durable remission through resection alone,^[23]^ as evidenced by Roka et al reporting 100% recurrence-free survival in 5 without nodal involvement patients after complete excision.^[15]^ For locally advanced cases, en bloc resection encompassing infiltrated organs is advocated to reduce local recurrence and improve long-term survival,^[13]^ with therapeutic lymphadenectomy indicated for both confirmed and suspected nodal metastases.^[27]^

However, the complex perithyroidal anatomy obligates extended resections in locally advanced ITTC, escalating surgical morbidity. Ebina et al requirement of total pharyngectomy for radical clearance exemplifies such challenges.^[28]^ Crucially, 38% of patients demonstrate tracheal invasion at diagnosis,^[13]^ necessitating resection-anastomosis^[12,26,29]^ with inherent anastomotic complication risks. Moreover, functionally critical structures frequently preclude R0 resection (resulting in R1/R2 status), compelling adjuvant radiotherapy to compensate for suboptimal surgery.^[8,30,31]^

Multidisciplinary team (comprising endocrine surgery, medical oncology, radiation oncology, and pathology) evaluation confirmed unresectable ITTC with circumferential tracheal invasion in this research. Incisional biopsy established the diagnosis, and the family declined palliative surgery. Given the extensive tumor bulk (>5 cm), we initiated chemotherapy or radiotherapy based on National Comprehensive Cancer Network (NCCN) Thymic Carcinoma Guidelines principles for unresectable bulky disease.

Emerging consensus recently supports radical radiotherapy as the primary treatment for unresectable ITTC,^[1,26,32,33]^ underpinned by its radiobiological similarity to thymic carcinomas where radical radiotherapy achieves >70% 5-year overall survival in selected cases.^[34–36]^ Petra et al documented CR in locally advanced ITTC after 70Gy tumor-bed irradiation plus 54Gy nodal irradiation.^[32]^ However, radiotherapy alone carries significant limitations. The foremost limitation is dose-dependent efficacy. Tsutsui’s case achieved initial remission with 45Gy but recurred at 10 years,^[13]^ indicating subtherapeutic dosing fails to eradicate radioresistant clones. For another, microscopic residual disease in sanctuary sites (e.g., retrosternal lymphatics) provides the pathological substrate for late recurrences.

Critically, experts highlighted that unresectable recurrence detected about 10 years after radiotherapy alone were the cause of eventual mortality.^[13]^ This failure pattern necessitates strategy escalation: either dose intensification (60–70Gy According to the NCCN guidelines of thymic carcinoma^[36–38]^) or concurrent chemotherapy to target sanctuary residues and micrometastases.

Up to now, there is limited evidence on chemotherapy for the treatment of unresectable ITTC. We selected the TP regimen as first-line induction chemotherapy based on the pathological similarity between ITTC and thymic carcinoma, notably the frequent presence of squamous differentiation (as evidenced by p40 and CK5/6 positivity in our case). This choice aligns with the NCCN guidelines for thymic carcinoma.

At present, chemotherapy has been mainly proven to be effective in patients with recurrent and metastatic ITTC.^[14]^ Various agents, including epirubicin, cisplatin, docetaxel, paclitaxel, carboplatin, etoposide, doxorubicin, cyclophosphamide, and nimustine. Similar to thymic carcinoma, ITTC seems to be sensitive to platinum. For instance, Abeni et al reported a CR in a recurrent ITTC patient treated with carboplatin and paclitaxel.^[39]^ Roka et al documented CR in an ITTC patient with pulmonary metastases following 6 cycles of palliative TP chemotherapy.^[15]^ Another case described a patient with lung metastases who responded well to both first-line (cisplatin, doxorubicin, vincristine, and cyclophosphamide [regimen]) and second-line (carboplatin and paclitaxel) chemotherapy.^[14]^ Conversely, ITTC appears insensitive to doxorubicin-based therapy.^[15,22,40]^ For example, Kakudo et al observed no clinical response in an ITTC patient with pulmonary metastases treated with doxorubicin, cyclophosphamide, and nimustine.^[22]^ while Roka et al reported a lack of response to an APC regimen (likely containing doxorubicin) in a different patient with liver metastases.^[15]^ In the absence of first-line chemotherapy outcomes for inoperable ITTC, we note that synchronous chemoradiation—employed against histologically analogous thymic carcinoma—achieves superior 5-year overall survival (67.7% vs 34.4%/33.3% with radiotherapy/chemotherapy alone).^[41–43]^ This therapeutic parallelism, coupled with our observed 40-month progression-free survival, supports the potential efficacy of combined modality therapy for ITTC.

Consistent with the reported activity of platinum-based regimens, our patient achieved tumor shrinkage and significant symptomatic relief after 5 cycles of TP chemotherapy. These findings collectively support the potential efficacy of the TP regimen for managing unresectable ITTC and warrant further investigation.

The pronounced chemosensitivity observed in this inoperable ITTC case, particularly to platinum-based regimens, may be attributed to the complex interplay between tumor biology and host immune response. Cisplatin enhances immune surveillance and promotes T cell activation through dephosphorylation signal transduction and activator of transcription 6, thereby enhancing the anticancer effect of the immune system.^[44,45]^ However, cisplatin has certain immunomodulatory effects, but it has limited effect in inducing immunogenic cell death (ICD), although cisplatin can trigger the release of high mobility group protein B1, but cannot effectively induce the exposure of calreticulin, which is one of the key signals of ICD, on the cell surface.^[44]^ Future studies could explore cisplatin derivatives or alternative chemotherapy agents to enhance their ICD-inducing capabilities.

From a clinical perspective, these findings have important implications for managing advanced ITTC. For patients achieving CR like ours, we recommended close surveillance with quarterly imaging and PD-L1 monitoring, as the high baseline expression suggested potential susceptibility to immune checkpoint inhibitors upon recurrence. For partial responders, we should propose intensifying treatment with chemoimmunotherapy combinations, particularly those incorporating PD-1/PD-L1 inhibitors. Moreover, targeted therapy based on the results of genetic testing is another viable option for subsequent treatment, with active trials of antitumor vascular agents like anlotinib available which was published in Chinese-language journals.

To our knowledge, this represents the first report demonstrating first-line platinum-based chemotherapy followed by chemoradiotherapy inducing durable complete remission (40-month PFS) in unresectable ITTC, thereby extending the NCCN thymic carcinoma paradigm to this rare entity. Through comprehensive molecular profiling and longitudinal response assessment, we established that: First, the multimodal regimen (induction chemotherapy followed by chemotherapy) achieved rapid tumor volume reduction even in PD-L1-high, tumor mutational load-low cases; Second, it is speculated that chemotherapy-induced ICD could overcome the immunosuppressive microenvironment characteristic of ITTC. These findings challenged the conventional paradigm of surgery-first approaches and propose chemotherapy as a viable standalone option for unresectable cases. Future multicenter trials should validate these results and explore chemoimmunotherapy combinations to further improve outcomes.

## Acknowledgments

Here, we sincerely thank all the authors of this study for their joint efforts and professional knowledge, which have provided important support and assistance for the completion of this study. Every author has put in hard work and enthusiastic investment in research design, data collection, writing, and review processes. Their cooperation and contribution are the key to the success of this study. Special thanks professor Tan and professor Xu, their respective professional knowledge and unique perspectives have provided valuable insights for this study. Without their support and cooperation, this study would not have been completed smoothly.

## Author contributions

**Conceptualization:** Bo Li.

**Data curation:** Bo Li.

**Funding acquisition:** Mingjin Xu.

**Investigation:** Yanchen Gong.

**Project administration:** Hui Feng.

**Resources:** Hui Feng.

**Software:** Xiaojun Wang.

**Supervision:** Xiaojun Wang, Xintong Wang, Ye Tan.

**Validation:** Xintong Wang.

**Visualization:** Xiaotong Huang.

**Writing – original draft:** Xiaotong Huang.

**Writing – review & editing:** Ye Tan.
